# In silico virtual screening of lead compounds for major antigenic sites in respiratory syncytial virus fusion protein

**DOI:** 10.1007/s42247-021-00213-6

**Published:** 2021-05-03

**Authors:** Shilu Mathew, Sara Taleb, Ali Hussein Eid, Asmaa A. Althani, Hadi M. Yassine

**Affiliations:** 1 Biomedical Research Center, Qatar University, Doha, 2713 Qatar; 2 College of Health and Life Sciences, Hamad Bin Khalifa University, Doha, Qatar; 3 College of Medicine, Qatar University, Doha, 2713 Qatar; 4 College of Health Sciences, Qatar University, Doha, 2713 Qatar

**Keywords:** In silico virtual screening, Respiratory syncytial virus, Fusion glycoprotein, Lead compounds, Antigenic sites

## Abstract

Human respiratory syncytial virus (RSV) is a leading ubiquitous respiratory pathogen in newborn infants, young children, and the elderly, with no vaccine available to date. The viral fusion glycoprotein (RSV F) plays an essential role in the infection process, and it is a primary target of neutralizing antibodies, making it an attractive site for vaccine development. With this in view, there is a persistent need to identify selective antiviral drugs against RSV, targeting the major antigenic sites on the F protein. We aimed to conduct a robust in silico high-throughput drug screening of one million compounds to explore potential inhibitors that bind the major antigenic site Ø and site II on RSV F protein, which are the main target of neutralizing antibodies (NAb). We utilized the three-dimensional crystallographic structure of both antigenic site Ø on pre-F and antigenic II on post-F to screen for potential anti-RSV inhibitors. A library of one million small compounds was docked to explore lead binders in the major antigenic sites by using virtual lab bench CLC Drug Discovery. We also performed Quantitative Structure-Activity and Relationship (QSAR) for the lead best binders known for their antiviral activity. Among one million tested ligands, seven ligands (PubChem ID: 3714418, 24787350, 49828911, 24802036, 79824892, 49726463, and 3139884) were identified as the best binders to neutralizing epitopes site Ø and four ligands (PubChem ID: 865999, 17505357, 24802036, and 24285058) to neutralizing epitopes site II, respectively. These binders exhibited significant interactions with neutralizing epitopes on RSV F, with an average of six H bonds, docking energy of − 15.43 Kcal·mol^−1^, and minimum interaction energy of − 7.45 Kcal·mol^−1^. Using in silico virtual screening, we identified potential RSV inhibitors that bind two major antigenic sites on the RSV F protein. Using structure-based design and combination-based drug therapy, identified molecules could be modified to generate the next generation anti-RSV drugs.

**Supplementary Information:**

The online version contains supplementary material available at 10.1007/s42247-021-00213-6.

## Introduction

Human respiratory syncytial virus (RSV) has been a key focus of the healthcare system worldwide and a high priority for vaccine development since it was first isolated in 1956 [1]. The virus is a major cause of lower respiratory tract infection (LRTI) in all age groups, leading to major clinical problems in young, elderly, and immunocompromised populations. In the USA alone, infection with RSV results in high hospitalization and annual mortality rates reaching 125,000 cases in children below the age of 5 [2]. With no vaccine nor treatment available, RSV continues to be a lead agent of infection-induced death and lower respiratory diseases in newborns, including bronchiolitis, pneumonia, and possibly wheezing and asthma later in life [3–6]. RSV (order: *Mononegavirales*; family: *Pneumoviridae*; genus: *Orthopneumovirus*) is a non-segmented, negative-sense RNA virus, encoding for 11 known proteins: five ribonucleocapsid, three surface proteins, and two non-structural and one inner envelope protein [7, 8]. Surface proteins, attachment (G), and fusion (F) are the main targets of neutralizing antibodies. F protein particularly is a challenging target for transforming irreversibly from a metastable (pre-F) to a stable structure (post-F) through the rearrangement of its refolding regions 1 and 2 (RR1 and RR2) at F1 peptide, located after F2 and p27 peptides [9]. Development of RSV vaccine has been unsuccessful, and previously developed vaccine was ineffective and in some cases resulted in enhanced-disease illness [10, 11]. In addition to supportive care, ribavirin and palivizumab are currently the only approved agents for RSV treatment and prophylaxis, respectively [12, 13]. Palivizumab is a monoclonal antibody targeting shared epitopes between both conformations of the fusion protein (pre-F and post-F) and is mostly given to high-risk infants but provides low levels of protection in treated patients (less than 50%) [14]. Recent advances in structural biology have enabled a better understanding of F protein, identifying pre-F glycoprotein as the main target for neutralizing antibodies (NAb) [15, 16].

Consequently, pre-F has become an attractive target for vaccine development and treatment intervention [11, 17]. However, how soon a safe and effective vaccine will be accessible to the public is still questionable. Accordingly, there is an urge to discover alternative antiviral drugs to control RSV infections. The pre-F structure holds several antigenic sites with promising neutralizing potencies, such as site Ø and site IV [15, 18–20], which can serve as targets for new inhibitors. Importantly, site Ø is on the top of the pre-F structure, occupying the amino acid (AA) residues 62–69 and 196–209, and binds to D25 and 5C4 mAbs [21]. Additionally, site II, situated between AA 255 and 275, is the target of monoclonal antibodies palivizumab and motavizumab and is a common neutralizing epitope between pre-F and post-F conformations. Previous studies attempted to screen for new RSV entry inhibitory molecules such as imidazopyridine derivatives, cyclopiazonic acid (CPA), benzimidazole-based compounds, GPAR-3710, and JMN3-003 [14, 22–24]. These diverse molecules showed a successful RSV entry inhibition through docking analysis, particularly when bound to active F antigenic sites and hydrophobic cavities. Yet, none of the aforementioned or nonmentioned inhibitors had fruitful outcomes in clinical trials. Notably, many of these compounds were tested before the stabilized pre-F structure was revealed.

Herein, we used CLC Drug Discovery Workbench 3.02 to virtually screen about a million compounds from different input libraries for their binding properties and kinetics to two major antigenic sites (site Ø and site II regions) on the F protein. According to a strict criteria program, we identified several molecules that bind these epitopes with a high number of hydrogen (more than six) bonds and minimum docking scores (negative value) [25, 26]. Identified molecules could be further modified and tested in vitro and in vivo as potential therapeutic agents against RSV.

## Materials and methods

### Preparation of F-glycoprotein structure

The 3D crystal structures of RSV pre-F in complex with 5C4 Fab (PDB ID: 5 W23, 2.85 Å of resolution; UniProtKB AC: A0A097PF39) and post-F trimeric protein (PDB ID: 3RRR, 2.85 Å of resolution; UniProtKB AC: A0A097PF39) protein were retrieved from the Data Bank [27]. The macromolecules were then refined with the H bond (HB) assignment (water orientations, at neutral pH), and energy was minimized with the Merck molecular force field 94 (MMFF94) force field [28]. The structure was then refined, making a minimization of the conformational energy to generate 3D molecule structures on imports [29]. The post-processing step was applied for small molecules with no rotatable bonds with an energy window of 5 Kcal/mol [30].

### Ligands

Input libraries of one million drugs were chosen from different sources for virtual screening. This includes 9270 anti-RSV tested active drug-like compounds reported from literature [31], 54,525 compounds from Chemical Entities of Biological Interest (CheBi) database [32], 50,000 compounds from Diverse p-library [33], 50,000 compounds from Natural database [34], 1000 compounds from MTiOpenScreen [35] and 550,000 compounds from PubChem chemical directory [36]. Ligands downloaded in simplified molecular-input line-entry system (SMILES) string were converted to Spatial Data File (SDF) format for ligand preparation. CLC Drug Discovery Workbench was applied to import ligands using the freely available program “Balloon,” which is used for the 3D structure generation [37].

### Active site prediction

**Fig. 1:**
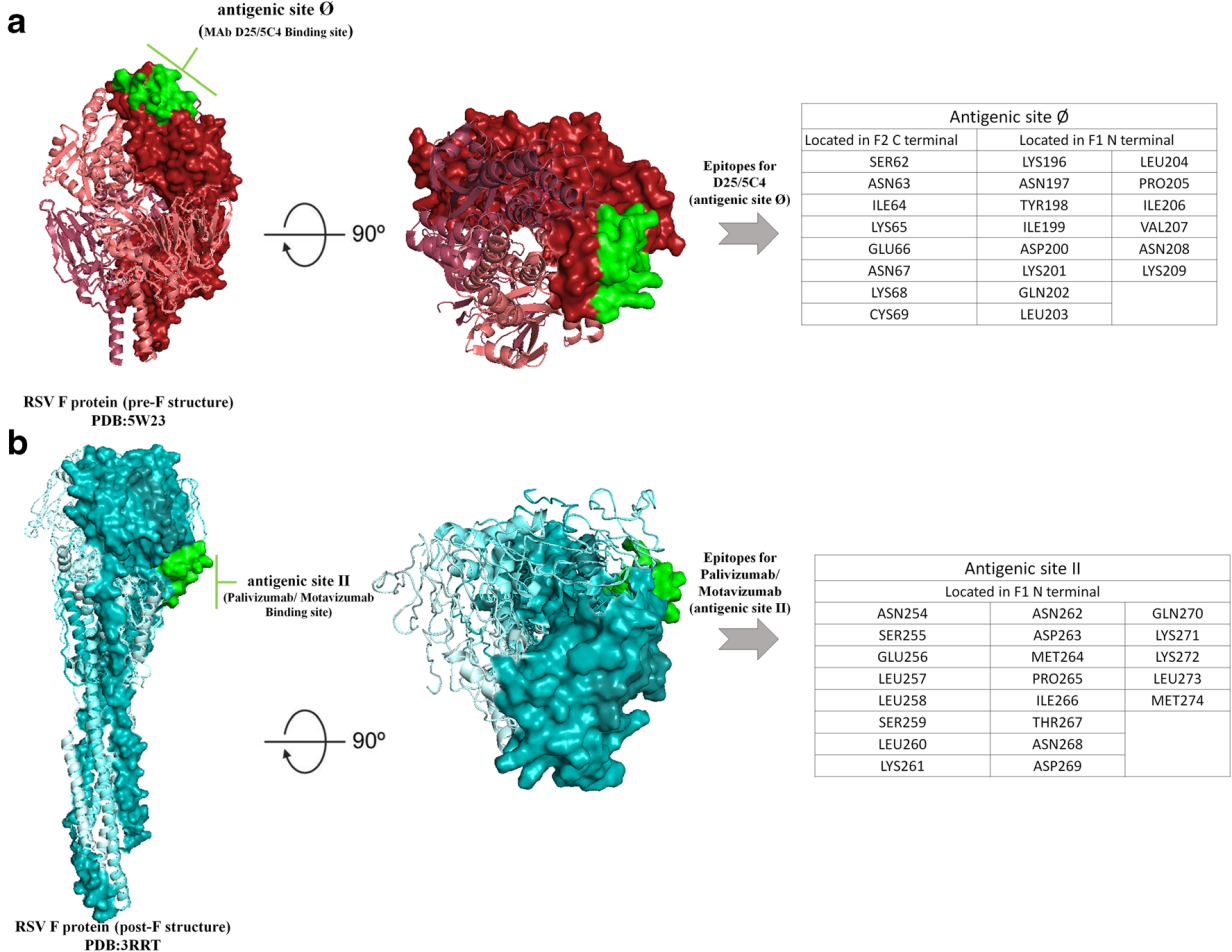
Structure and neutralizing epitopes on RSV F protein. **a** The left panel shows the overall structure of the pre-F structure confirmation of RSV F protein (PDB: 5 W23) in a burgundy color and post-F structure (PDB: 3RRT) in cyan color. Two protomers are shown as a cartoon in both representation, and one protomer highlighted with their contact residues is shown as surface representation. Both the contact residues in antigenic site Ø and II are shown in green color (surface). **b** The middle panel shows the structure turned 90° and shown looking down at the viral membrane with an antigenic epitope of D25/5C4 in pre-F structure and epitopes of palivizumab/motavizumab binding site in post-F structure. **c** The right panel denotes tables with contact residues in antigenic site Ø and II

Active site regions for both site Ø and site II were based on contact residues targeted by NAb on both pre-F and post-F glycoproteins. The contact residues of site Ø, as determined by stabilized pre-F structure by mAb5C4, include AA residue 62–69 and 196–209 AA [38]. In specific, this includes residues loop between β2-strand and α1-helix (SER62, ASN63, ILE64, LYS65, GLU66, ASN67, LYS68, CYS69) located in F2 C-terminal and residues from α4-helix (LYS196, ASN197, TYR198, ILE199, ASP200, LYS201, GLN202, LEU203, LEU204, PRO205, ILE206, VAL207, ASN208, and LYS209) located in F1 N-terminal [38]. On the other hand, the contact residues of site II-specific epitope (palivizumab epitope) comprises residues located at α6 and α7 helix 255 to 275 AA (ASN254, SER255, GLU256, LEU257, LEU258, SER259, LEU260, LYS261, ASN262, ASP263, MET264, PRO265, ILE266, THR267, ASN268, ASP269, GLN270, LYS271, LYS272, LEU273, and MET274) [39]. Contact residues on antigenic of site Ø and site II are depicted in Fig. 1**.**

### Virtual screening with CLC Drug Discovery Workbench

CLC Drug Discovery Workbench was then used to generate ten best poses for each conformation of the best docking energy (DE) based on scoring functions [28]. Docking wizard was used by applying the default MolDock optimizer algorithm with the following docking parameters, including 200 number of runs, maximum iterations 2000, crossover rate 0.90, scaling factor 0.50, and RMSD thresholds for similar cluster poses were set as 1.00 [28]. The best-ranked compounds were selected based on HB (more than six HB), docking score (DS) (minimum negative score), and interaction energy (IE)/binding affinity (BA) with smaller dissociation constants (Kd) indicating better binding. Additional docking tool YASARA (Yet Another Scientific Artificial Reality Application), an AutoDock-based tool for molecular docking and virtual screening, was used for analyzing dissociation constant (Kd) and binding energy of the docked complexes [40, 41].

### Pharmacokinetics structure-activity relationship

The selected best binders were considered to evaluate their physicochemical properties and their relation with biological activities [42]. The Quantitative Structure-Activity Relationships (QSAR) predicts the compound’s biological expected response according to its chemical structure [42]. VEGA-QSAR is an independent Java-based web program that predicts QSAR properties and screens similar compounds in a read-across strategy. Mutagenicity (Ames test) CONSENSUS model 1.0.3, carcinogenicity model (CAESAR) 2.1.9, carcinogenicity inhalation classification model (IRFMN) 1.0.0, developmental toxicity model (CAESAR) 2.1.7, skin sensitization model (CAESAR) 2.1.6, hepatotoxicity model (IRFMN) 1.0.0, ready biodegradability model (IRFMN) 1.0.9, and LogP Prediction [Log Units] models were evaluated by publicly well-known open and commercial QSAR prediction software package VEGA [43]. Spatial data file (SDF) files were used as input formats of the 2D structures of the ligands from PubChem. Results evaluated by VEGA models could be adequate to determine the physicochemical characteristics of the selected compounds [42]

## Results

### Virtual screening and selection of hit leads for pre-F (site Ø) and post-F proteins (site II)

**Fig. 2:**
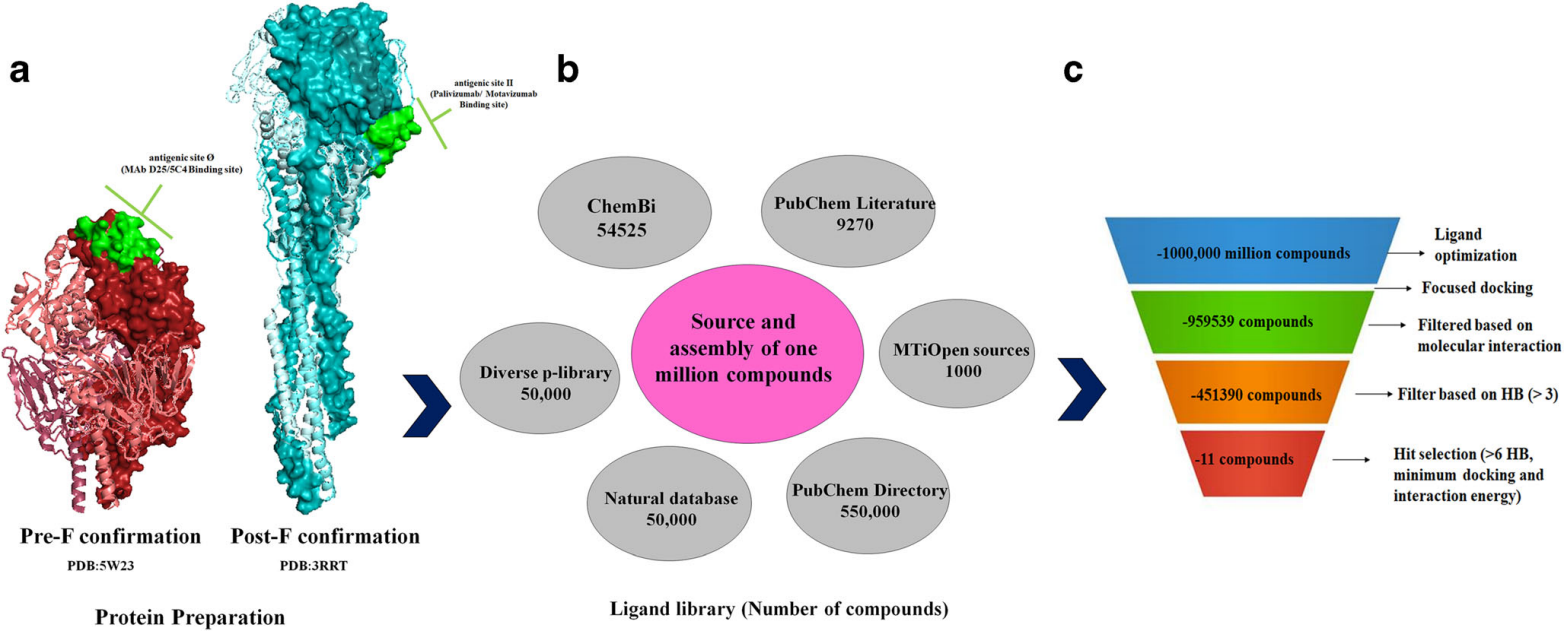
The overall workflow of the structure-based virtual screening of one million compounds library against RSV F antigenic sites Ø and site II. **a** Three-dimensional X-ray crystallography structure of RSV F protein structure in pre-F and post-F confirmations. The protein structures were prepared using CLC Drug Discovery Workbench, and the selected antigenic site Ø and site II are represented in green and pink space- filled CPK structures, respectively. **b** Library selection from different sources comprising 1,000,000 million compounds in total. The compounds were downloaded in SDF format and imported as PDB format for virtual screening. **c** Denotes virtual screening workflow, which includes ligand preparation and initial filtration by using CLC Drug Discovery Workbench. The compounds with molecular interaction with a cut-off HB score of 6 and with a minimum docking and interaction energy (negative score) were selected as hit compounds

In order to efficiently screen and identify the best inhibitors against major antigenic sites, Ø and II, we employed a multistep screening framework. X-ray crystallography structure of pre-F harboring antigenic site Ø and post-F harboring antigenic site II were prepared as per the protein preparations mentioned in CLC Drug Discovery Workbench [28]. Information about the targeted epitopes was collected from the available literature [39]. Figure 2 illustrates the overall workflow of the structure-based virtual screening of one million compounds (from different sources) against RSV F antigenic sites. Prior to performing a virtual screening, a selection of the chemical library of one million compounds was applied by considering the Lipinski rules [44]. Furthermore, the following multistep strategy was employed to sort hit compounds. Firstly, compounds that showed no interaction with the active sites in site Ø and site II were excluded. Secondly, compounds that formed at least six HB with a minimum number of IE and DS were selected as the best binders.

To lessen the inconsistency of the docked results, we performed repeated docking analyses using YASARA [40]. Fig. 2 represents filtration and distribution of ~one million screened compounds over a range of DS (scores are in Kcal/mol), HB interaction, and IE/BA scores (scores are in Kcal/mol). Through the computational screen, we propose the best binders to have high HB binding with lower the BA adequate high IE and binding score. Using these criteria, a minimal number of seven chemical compounds were identified as suitable binders of antigenic site Ø and four compounds as good binders of antigenic site II.

### The predicted hit compounds of antigenic site Ø of RSV pre-F protein

**Fig. 3:**
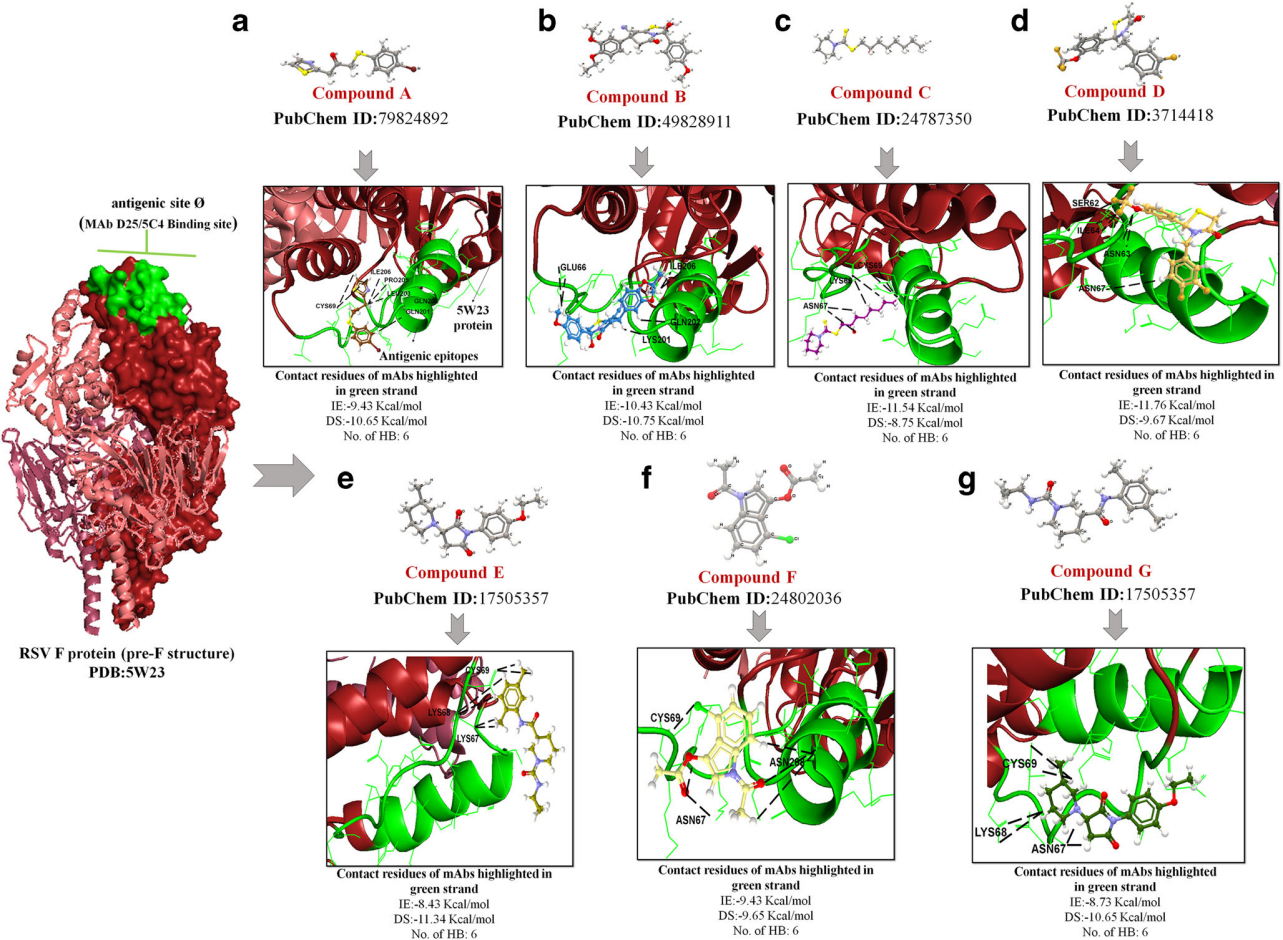
Computational docking confirmation of the seven best binders to antigenic site Ø on pre-F protein. The analysis was done with CLC Drug Discovery Workbench while considering the important parameters, including DS, IE, and HB interaction. The left panel shows the overall structure of the pre-F structure confirmation of RSV F protein (PDB: 5 W23) in burgundy color with major antigenic epitopes in site Ø shown as surface (green color). Green colored surface denotes a selective grid generated by CLC Drug Discovery Workbench for focused binding. The right panel depicts the binding characteristics of seven small molecules to their target. All compound were retrieved from PubChem including **a** PubChem ID:79824892, **b** PubChem ID:49828911, **c** PubChem ID:24787350, **d** PubChem ID:17505357, **e** PubChem ID:17505357, **f** PubChem ID:24802036, and **g** PubChem ID:17505357 and are shown as 2D structure. Burgundy ribbon-like structure represents a 5 W23 protein structure, and the green ribbon structure denotes the antigenic epitopes in site Ø. The anchored HB between the compound and site Ø epitope is shown in as black color

Using the computational docking approach and targeting antigenic site Ø of the pre-F, seven best binders exhibited the maximum number of H bond (all had six predictable HB) interactions with minimum BA score best electrostatic interaction. These lead molecules include PubChem ID: 79824892 (compound A), 49828911 (compound B), 24787350 (compound C), 3714418 (compound D), 3139884 (compound E), 24802036 (compound F), and 49726463 (compound G). The binding characteristics of the seven molecules, as well as mAb D25 to site Ø on Pre-F, are summarized in Table 1**.** The docking conformations of the abovementioned lead binders are illustrated in Fig. 3. Compound D exhibited the best atomic interaction with residues in SER62, ASN63 (2), ILE64 (2), and ASN67 (1), with a minimum IE of − 11.76 Kcal/mol and a DS of − 9.67 Kcal/mol (Fig. 3d). Compounds B and C recorded second best IE with an average of − 10.98 Kcal/mol (Fig. 3 b and e). Compound B showed potential interaction with ILE206 (2), GLU66 (2), GLN202 (1), and LYS201 (1) with a DS of − 10.75 Kcal/mol. Furthermore, compound **C** anchored with CYS69 (2), LYS68 (2), and ASN67 (2) with a DS of − 8.75 Kcal/mol. Apart from their efficient interaction, we observed that compounds A, F, and B complexed different orientation binding modes (or mechanisms) compared to the rest of the molecules. More precisely, the molecules, as mentioned above, anchored HB with the α4-helix (F1 residues: 196–210) and F2 C-terminal (AA 62–69) between β2-α1 loop, respectively. However, compounds A and B extended surplus interactions at α4-helix residues (F1 N-terminal) compared to F2 C-terminal in antigenic site Ø. Compound A anchored in strand α4-helix at F1 N-terminal to residues ILE206 (1), PRO205 (1), GLN205 (1), and LEU203 (1) and at F2 C-terminal to CYS69 (2) (Fig. 3a). Likewise, potential interactions of compound B took place at F1 N-terminal residues ILE206 (2), GLN202 (1), and LYS201 (1) and F2 C-terminal residue GLU66 (2) (Fig. 3b). Moreover, compound F established an interaction at site ASN208, but with an intermolecular interaction of three H bonds **(**Fig. 3f). Besides, compound F established a contact with residues CYS69 (1) and ASN67 (2) at a minimum IE of − 9.43 Kcal/mol and a DS of − 9.65 Kcal/mol. Conversely, lead compounds D, E, and G formed favorable interactions only in contact with site Ø, between β2-α1 loop residues (AA 62–69). In detail, the predicted binding pose of compounds E, G, and C demonstrated intermolecular HB with residue LYS68 in the loop region, which is recognized as one of the D25-targeted quaternary epitopes [20]. Similarly, compound D interacted with F2 residue ASN63, which is also recognized as one of the D25-targeted quaternary epitopes (Fig. 3d). The average IE and DS of compounds E, G, and C were − − 9.56 Kcal/mol IE and − 10.58 Kcal/mol (Fig. 3 e, g, and c). Notably, compounds E, F, and G extended HB interactions with residue CYS69, which is proposed to link the C-terminal F1 and the N-terminal F2 subunits in a single disulfide bridge, and play important roles in the folding and functioning of the molecule [45]. Another noteworthy observation was the interaction of these compounds (compounds C, D, E, F, and G) specifically to the ASN67 AA region located in α4-helix and the β2-α1 loop with an average IE of − 9.56 Kcal/mol and DS of − 10.58 Kcal/mol. In addition to their efficient binding, these lead compounds anchored several linear and discontinuous residues located around the antigenic site Ø.

**Table 1:** In silico screening analysis between libraries of one million compounds interacted with antigenic site Ø region and their intermolecular docking values presented with interaction energy, H bond energy, docking score, number of H bond interaction, and the interacting residues

S. No.	Compound	PubChem ID	Interaction energy (Kcal/mol)	Number of H bonds	Residue interactions	Docking score (Kcal/mol)
1	A	79824892	− 9.43	6	CYS69(2), ILE206(1), PRO205(1), GLN201(1), LEU203(1)	− 10.65
2	B	49828911	− 10.43	6	ILE206(2), GLU66(2), GLN202(1), LYS201(1)	− 10.75
3	C	24787350	− 11.54	6	CYS69(2), LYS68(2), ASN67(2)	− 8.75
4	D	3714418	− 11.76	6	SER62(1), ASN63(2), ILE64(2), ASN67(1)	− 9.67
5	E	3139884	− 8.43	6	ASN67(2), LYS68(2), CYS69(2)	− 11.34
6	F	24802036	− 9.43	6	ASN208(3), CYS69(1), ASN67(2)	− 9.65
7	G	49726463	− 8.73	6	LYS68(2), CYS69(2), ASN67(2)	− 10.65
Control MAbs						
8	D25	-	− 3.68	11	SER62(1), ASN63(1), ILE64(1), LYS65(1), GLU66(1), ASN67(1), LYS68(1), TYR198(1), ILE199(1), ASP200(1), LYS201(1)	− 2.78

### The predicted hit compounds of antigenic site II of RSV post-F protein

**Fig. 4:**
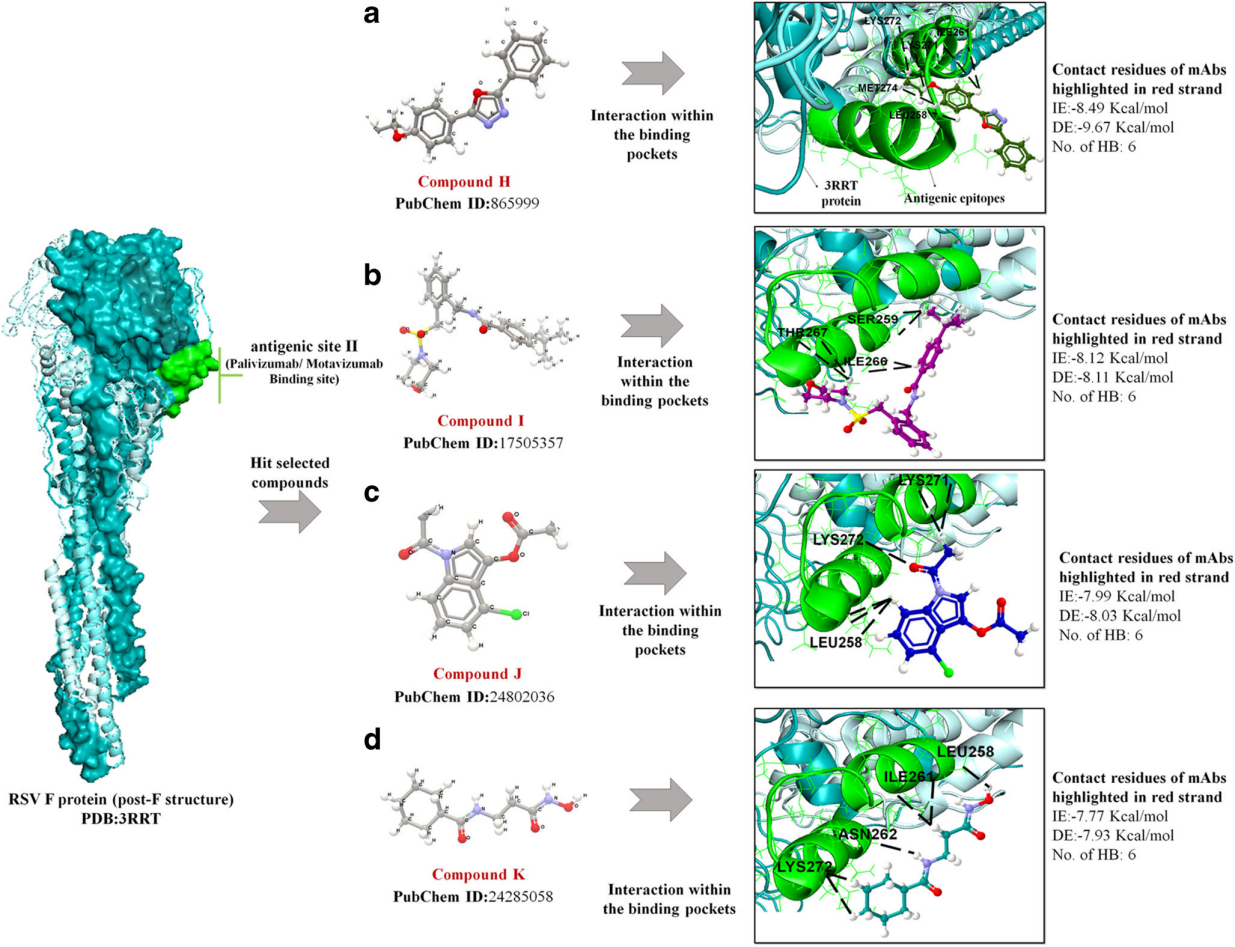
Computational docking confirmation of the four best binders to antigenic site II RSV F protein. The analysis was done with CLC Drug Discovery Workbench. The analysis was done by considering the important parameters, including DS, IE, and HB interaction. **a** The left panel shows the post-F structure of RSV (PDB:3RRR) in cyan color with major antigenic epitopes in site II is shown as a green surface. Green colored surface denotes a selective grid generated by CLC Drug Discovery Workbench for focused binding. **b** The middle panel shows the best four binders screened considering parameters, including DS, IE, and HB interaction. The following are the compounds selectively: **a** PubChem ID:865999, **b** PubChem ID:17505357, **c** PubChem ID:24802036, and **d** PubChem ID:24285058. **c** The right panel denotes interaction within the binding pockets. The cyan colored ribbon-like structure represents 3RRR protein structure, and the green ribbon structure denotes the antigenic epitopes in site Ø. The anchored HB between the compound and site Ø epitope is shown in as black color

Using a computational docking approach targeting antigenic site II of the post-F protein, we next evaluated the remaining four best binders interacted with a maximum number of HB (all had six predictable HB) interactions and a minimum BA score. These four lead binders include PubChem ID: 865999 (compound H), 17505357 (compound I), 24787350 (compound J), and 24285058 (compound K). The docking conformations of the lead binders H–K are illustrated in Fig. 4. Compound H exhibited the best atomic interaction with residues MET274, LYS272, LYS271, ILE261 (2), and LEU258 (1), with a minimum IE of -8.49 Kcal/mol and a DS of − 9.67 Kcal/mol (Fig. 4a). Compound I ranked as a second best binder with six HBs: THR267 (2), ILE266 (1), and SER259 (2) of the F1 N-terminal in the α6 and α7 helix (Fig. 4b). Interestingly, compound I anchored with common unique residues to palivizumab binding epitopes. Moreover, all the best binders adopted maximum HB in the α6 and α7 conformation compared to the loop region. All three compounds H, J, and K extended an HB interaction with LYS272 residue, located at F1 N-terminal (α6 and α7 helix). LYS272 is identified as AA of escape mutant that changes preferably (to GLU272 or THR272) to avoid palivizumab, mAb1129 mAb binding (Fig. 4 a, c, and d) [46]. On average, these compounds formed an average of − 8.12 Kcal/mol IE and − 8.57 Kcal/mol DS (Fig. 4d). Compound K anchored with AA residues ILE261 (2), LEU258 (1), LYS272 (2), and ASN262 (1) in the F1 N-terminal. The binding characteristics of the four molecules, as well as mAb palivizumab to site II on post-F, are summarized in Table 2.

**Table 2:** In silico screening analysis between libraries of one million compounds interacted with antigenic site II region and their intermolecular docking values presented with interaction energy, H bond energy, docking score, number of H bond interaction, and the interacting residues

S. No.	Compound	PubChem ID	Interaction energy (Kcal/mol)	Number of H bonds	Residue interactions	Docking score (Kcal/mol)
1.1.	H	865999	− 8.49	6	LEU258(1), ILE261(2), LYS271(1), LYS272(1), MET274(1)	− 9.67
2.2.	I	17505357	− 8.12	6	SER259(2), ILE266(2), THR267(2)	− 8.11
3.3.	J	24802036	− 7.99	6	LEU258(3), LYS271(2), LYS272(1)	− 8.03
4.4.	K	24285058	− 7.77	6	LEU258(1), ILE261(2), ASN262(1), LYS272(2)	− 7.93
Control mAbs						
5.5.	Palivizumab	46506637	− 0.53	12	SER255 (1), GLU256(1), LEU258(1), SER259(1), LYS261(1), ASN262(1), ILE266(1), THR267(1), ASN268(1), ASP269(1), LYS271(1), LYS272(1)	− 1.56

### VEGA-QSAR profiling of screened products

All the best binders were then screened through the QSAR model for their biological activities, including mutagenicity, sensitivity, biodegradability, toxicity, and carcinogenicity. Results evaluated by QSAR models denoted that 90% of the lead binders were non-mutagen and non-toxicant. Hepatotoxicity model (IRFMN) 1.0.0 toxicant compound included compound A, compound B, and compound D. Toxicant compounds included all identified compounds, except B and E, exhibited sensitivity to skin sensitization model (CAESAR) 2.1.6. Out of the seven compounds, A, B, and D had a positive prediction for the hepatotoxicity model. Compounds C, E, H, and I were predicted to be ready biodegradable compared to the remaining others. Predicted values log *P* value of the lead binders ranged from 0.25 to 5.99 log units. This value was observed higher for all the lead compounds except for compound K, which had a log *P* value less than 1.00 log unit. Predicted properties for the best binders to site Ø and site II for various models are summarized in supplementary information Table S1 and Table S2.

## Discussion

RSV is still the leading cause of lower respiratory tract disease in infants, to which neither vaccines nor treatments are available [47]. Further, RSV affects all age groups [48] and causes repeated infections without significant changes in the antigenic sites [49, 50]. Therefore, there is a significant need to identify or develop efficacious therapeutics, including novel small molecules, to control RSV infections.

The fusion (F) protein is a class I fusion glycoprotein and has been identified as a major target for antiviral drugs and vaccine development [49, 51]. Until recently, the development of an efficient vaccine has been hurdled by the limited understanding of the conformational rearrangement between metastable pre-F and stable post-F [52, 53]. Nonetheless, solving the crystal structural of the pre-F protein revealed major neutralizing epitopes, additional to those present in the post-F conformation [20]. Among these, site Ø represents the major target for neutralizing antibodies, accounting for 35% and 47% of the overall response in RSV-A and RSV-B, respectively [19]. Additionally, site II is present on both conformations and is the target for the traditionally prophylactic antibody palivizumab [20, 54]. However, there are several limitations in using palivizumab [55]. For example, it is recommended to only treat premature infected infants, but it is not advised to treat those with congenital heart disease and other selected populations [54]. On the other hand, the recently structurally defined antigenic site Ø on pre-F has a neutralization potency 10- to 100-fold greater than palivizumab [38]. Considering all the above, we designed this in silico analysis to screen for inhibitors that can interfere with both sites and could be potentially tested as antiviral drugs in the future.

To do so, we used a similar approach to what we have recently reported with influenza [56] and ran a high-throughput computational screening of one million selected by using the CLC Drug Discovery Workbench. Firstly, compounds that showed no interaction with the active sites in site Ø and site II in the initial screening were excluded. Secondly, compounds that formed at least six HB with a minimum number of IE and DS were selected as the best binders. Using this method, we selected the ten best poses for each conformation of the best docking energy (DE) based on scoring functions [57]. Using these criteria, we identified seven and four hit candidates that target RSV site Ø (pre-F) and site II (post-F), respectively. All the best binders were then screened through the QSAR model for their biological activities, including mutagenicity, sensitivity, biodegradability, toxicity, and carcinogenicity. Results evaluated by QSAR models denoted that 90% of the lead binders were non-mutagen and non-toxicant. Our analysis outperforms previously reported approaches [58] by using structurally defined models to target specific neutralizing epitopes.

In terms of site Ø, the seven compounds reflected the potential to bind with a minimum of six HB and less than − 11.76 Kcal/mol IE. These include PubChem ID: 79824892 (compound A), 49828911 (compound B), 24787350 (compound C), 3714418 (compound D), 3139884 (compound E), 24802036 (compound F), and 49726463 (compound G). Among all, only compounds A and B extended more HB in F1 N-terminal compared to the F2 C-terminal of the pre-F protein structure. More specifically, compound A interacted with ILE206 (1), PRO205 (1), GLN205 (1), and LEU203 (1) in F1 N-terminal and CYS69 (2) in F2 C-terminal with a minimum IE of − 9.43 Kcal/mol and a DS of − 10.65 Kcal/mol. Likewise, compound B interacted with residues ILE206 (2), GLN202 (1), and LYS201 (1) in F1 N-terminal and GLU66 (2) in F2 C-terminal, with minimum IE of − 10.43 Kcal/mol and a DS of − 10.75 Kcal/mol. On the other hand, the rest of the lead binders to site Ø (compounds C, D, E, and G) formed favorable interactions among residues in the loop between β2-strand and α1-helix loop in F2 C-terminal (AA 62–69). These bindings mimic the binding of D25 and 5C4 that buries a high fraction of the accessible surface area on the α4-helix, including the majority of the accessible surface area [27]. Biologically, the interior position of the F2 C-terminus is suggested to play a role in triggering the prefusion F conformation [59]. Another noteworthy observation was the interaction of compounds C, D, and E to ASN67 residue, which is located between the α4-helix and the β2-α1 loop with an average interaction energy (IE) of − 9.56 Kcal/mol and docking score (DS) of − 10.58 Kcal/mol. Such interaction has been demonstrated to influence the movement of α4-helix (F1 AA 196 to 210), where RR1 is overlapping (AA 137 to 216), thereby arresting and preventing the fusion peptide from refolding [9]. Residue ASN67 in the β2-α1 loop has been selected for site-directed mutagenesis for being less antigenically important than the charged residues at positions 65, 66, and 68 [9]. The ASN67 side chain does not have a fixed position and can reach α1- and α4-helix, forming a three-helices bundle with α5. Therefore, the interaction of lead molecules at ASN67 hydrophobic residue may prevent α4-helix from moving and hanging, which could have implications on improving the interaction with α1 and α4 that could stabilize the apex region of the protein [9].

Following the same approach, we identified four lead molecules that exhibit promising anti-RSV-site II activity. These include PubChem ID: 865999 (compound H), 17505357 (compound I), 24787350 (compound J), and 24285058 (compound K). Importantly, compounds H, J, and K extended HB interaction at residue LYS272, which has been identified as a vulnerable site to escape palivizumab neutralization [46]. Likewise, the same compounds extended other HB interactions with residue LEU258, another crucial site for AA alternation to escape palivizumab neutralization [60]. Additional interactions were also observed with residue ILE261, adjacent to the β2-α1 loop region. For instance, compound K interacted with residue N262, which is reported as a mutation-sensitive residue, rendering RSV resistant to neutralization by palivizumab, as shown in cell culture and cotton rat models [61].

Collectively, our identified drug-like molecules are found to make strong HB (in addition to hydrophobic interactions) with known crucial active residues of antigenic sites Ø and II. Accordingly, binding of these molecules to F protein is likely to neutralize the virus through both sites, potentially disrupt its conformational change needed for viral internalization through common residues of site Ø with RR1, and possibly recognize escape mutations at site II (better than palivizumab). Interestingly, lead compounds A–G, interacting with site Ø, displayed lower IE and DS than the highly potent D25 mAb. Similarly, the lead compounds H–K showed lower ID and ES scores in their potential binding to site II. These observations probably suggest that each of the identified compounds could have a high binding affinity to its corresponding antigenic sites Ø or II. Displaying these molecules in a multivalent format (nanoparticle or so) could further enhance their binding affinity [62–65]. Importantly, these small molecules have a better capacity to reach and bind their target without being very immunogenic [66]. Still, these assumptions require further validations using in vitro and in vivo experimentations.

A recent computational screening study from Kamal Kant et al. identified natural phytochemicals such as rutin, schaftoside, and apigenin as potential anti-RSV drugs. Although these phytochemicals compounds were also screened in our study, they displayed limited interaction with both site Ø- and II-targeted epitopes [67]. Accordingly, these compounds would be binding somewhere else on the F protein, away from the crucial neutralizing epitopes. Interestingly, a previous study by Aurelio Bonavia et al. demonstrated a broad-spectrum activity of natural phytochemicals that exceeds RSV to other viruses such as influenza virus and HCV [68]. The broad-spectrum activity of these natural compounds highly suggested interruption of de novo pyrimidine biosynthesis (essential for cell survival), a common cellular pathway involved in their mechanism of action against positive- and negative-sense RNA, and retroviruses. In vitro, these inhibitors had a toxic effect on dividing B and T cells, but not on primary human bronchial epithelial cells [68].

Using a similar approach, Cancellieri et al. also followed a computer-aided approach to screen a library of “small fragments series of zinc-reacting compounds” as potential inhibitors of RSV replication [58]. This study demonstrated a library of ∼ 12,000 zinc-chelating moieties, along with 30 compounds designed sharing a common dithiocarbamate moiety, which was prepared to target the zinc finger motif of the RSV M2-1 protein [69]. The most active compounds from Cancellieri et al. were also considered in this study, but results did not show any potential interactions. This may be due to the chemical structure of the compounds that specifically target zinc protein M2-1 in RSV that is involved in the control of viral polymerase processivity. The above study by Cancellieri et al. was based on a recent report by Boukhvalova et al. that demonstrated that infectivity of retroviruses such as HIV-1 and MuLV can be abrogated by compounds targeting zinc finger motif in viral nucleocapsid protein (NC), involved in controlling virus infectivity and the processivity of reverse transcription. Although RSV is a member of the different viral family, it was possible that zinc finger-reactive compounds that inactivate retroviruses would have a similar effect against RSV by targeting RSV M2-1 protein [69].

In summary, the above observations spotlight an effective mechanism of HB interaction to the defined antigenic sites, which has implications for their mechanisms of neutralization. Among one million tested ligands, seven ligands (PubChem ID: 3714418, 24787350, 49828911, 24802036, 79824892, 49726463, and 3139884) were identified as the best binders to neutralizing epitopes site Ø and four ligands (PubChem ID: 865999, 17505357, 24802036, and 24285058) to neutralizing epitopes site II. Results evaluated by QSAR models also denoted that 90% of the lead binders were non-mutagen and non-toxicant. These binders exhibited significant interactions with neutralizing epitopes on RSV F, with an average of six H bonds, docking energy of − 15.43 Kcal·mol^−1^, and minimum interaction energy of − 7.45 Kcal·mol^−1^. The binding of these molecules to F protein is likely to neutralize the virus through both sites, potentially arresting and preventing the fusion peptide from refolding needed for viral internalization. The interaction of the best binders may lead to the disruption of pre-F conformations’ crucial functions, thereby inhibiting RSV infectivity. Also, the screened best binders could be provided in the form of combinational therapy to treat high-risk patients of all age groups broadly**.** Although further studies are required to prove the mechanism of action of these compounds, these results represent an auspicious starting point for the development of a novel class of RSV inhibitors.

## Supplementary Information

ESM 1 (DOCX 19.7 KB)

ESM 2 (DOCX 194 mb)

## Data Availability

Data are available as a supplementary file and video file.
