# Supplementary material for: In silico virtual screening of lead compounds for major antigenic sites in respiratory syncytial virus fusion protein
Source: Emergent Mater. 2021 May 3;5(2):295–305. doi: 10.1007/s42247-021-00213-6 (PMC8090912; doi:10.1007/s42247-021-00213-6)

**Supporting Information- S Tables**

Table 1: QSAR predicted values and their applicability domain analysis for various models in site Ø

| **QSAR Models** | **Compound G** | **Compound F** | **Compound E** | **Compound D** | **Compound C** | **Compound B** | **Compound A** |
| --- | --- | --- | --- | --- | --- | --- | --- |
| Mutagenicity (Ames test) CONSENSUS model 1.0.3 |  |  |  |  |  |  |  |
| Carcinogenicity model (CAESAR) 2.1.9 |  |  |  |  |  |  |  |
| Carcinogenicity inhalation classification model (IRFMN) 1.0.0 |  |  |  |  |  |  |  |
| Developmental Toxicity model (CAESAR) 2.1.7 |  |  |  |  |  |  |  |
| Skin Sensitization model (CAESAR) 2.1.6 |  |  |  |  |  |  |  |
| Hepatotoxicity model (IRFMN) 1.0.0 |  |  |  |  |  |  |  |
| Ready Biodegradability model (IRFMN) 1.0.9 |  |  |  |  |  |  |  |
| LogP Prediction [Log Units] |  |  |  |  |  |  |  |


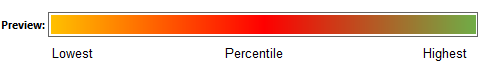


Table 2: QSAR predicted values and their applicability domain analysis for various models in site II

| **QSAR Models** | **Compound I** | **Compound H** | **Compound K** | **Compound J** |
| --- | --- | --- | --- | --- |
| Mutagenicity (Ames test) CONSENSUS model 1.0.3 |  |  |  |  |
| Carcinogenicity model (CAESAR) 2.1.9 |  |  |  |  |
| Carcinogenicity inhalation classification model (IRFMN) 1.0.0 |  |  |  |  |
| Developmental Toxicity model (CAESAR) 2.1.7 |  |  |  |  |
| Skin Sensitization model (CAESAR) 2.1.6 |  |  |  |  |
| Hepatotoxicity model (IRFMN) 1.0.0 |  |  |  |  |
| Ready Biodegradability model (IRFMN) 1.0.9 |  |  |  |  |
| LogP Prediction [Log Units] |  |  |  |  |


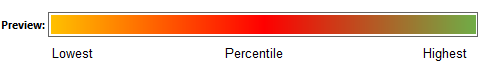

Supplement: Supplementary file 1 — (DOCX 19.7 KB) [file 42247_2021_213_MOESM1_ESM.docx]
